# Comparison of Adhesive Tape Impression Cytology, Hair Plucks, and Fungal Culture for the Diagnosis of Dermatophytosis in Dogs and Cats

**DOI:** 10.3390/vetsci10030183

**Published:** 2023-02-28

**Authors:** Pavlina Bouza-Rapti, Anastasia Karafylia, Androniki Tamvakis, Rania Farmaki

**Affiliations:** 1 Companion Animal Clinic, Medicine Unit, School of Veterinary Medicine, Faculty of Health Sciences, Aristotle University of Thessaloniki, 54627 Thessaloniki, Greece; 2 Laboratory of Ecology and System Dynamics, Department of Marine Sciences, University of the Aegean, 81100 Mytilene, Greece

**Keywords:** dermatophytosis, diagnosis, adhesive tape impression cytology, skin, dog, cat

## Abstract

**Simple Summary:**

The purpose of this study was to evaluate the sensitivity of adhesive tape impression (ATI) cytology for the diagnosis of dermatophytosis in dogs and cats and to compare this test with the most commonly used diagnostic tests: microscopic examination of plucked hairs and fungal culture. Diagnosis of dermatophytosis can be challenging in some cases, especially in dogs with kerions, and at least two tests should be used for diagnosis since no single test can be identified as the “gold standard”. The present study supports this conclusion, as in comparison between the three diagnostic tests in dogs and cats with dermatophytosis no significant difference was found, apart from dogs with kerion. In this group of dogs, ATI cytology and fungal culture had the same sensitivity that was higher than hair plucks, and hair plucks were significantly less sensitive than fungal culture. Additionally, adhesive tape preparations can be superior to other diagnostic tests in cats. ATI cytology is easy to perform, inexpensive, noninvasive, minimally stressful for the animals, and can be a useful in-house diagnostic test for dermatophytosis. However, more studies with larger populations should be conducted to further evaluate the sensitivity of this diagnostic test in dermatophytosis.

**Abstract:**

Rapid diagnosis of dermatophytosis is essential for early treatment induction and prevention of spreading to other animals and humans. No single diagnostic test is identified as the “gold standard”. The purpose of the study was to evaluate the sensitivity of adhesive tape impression (ATI) cytology in dermatophyte identification and to compare three diagnostic tests for dermatophytosis. Thirty dogs, with alopecia (*n* = 19) or kerion (*n* = 11), and fifteen cats with alopecia were included in the study. Dermatophytosis was diagnosed with tape preparations in 82.2% (37/45) of cases, while with hair plucks in 66.7% (30/45) and fungal culture in 80% (36/45). In kerions, tape preparations and fungal culture had the same sensitivity (10/11, 90.9%) that was higher than that of hair plucks (4/11, 36.4%). The sensitivity was higher in cats than in dogs with alopecia for all tests, 80% versus (vs.) 73.7%, 86.7% vs. 68.4%, and 93.3% vs. 68.4% for cats and dogs for hair plucks, fungal culture, and tape preparations, respectively. No significant difference was found between the three tests, except for dogs with kerion. Hair plucks were less sensitive than fungal culture in kerions (*p* = 0.041), while in comparison with tape preparations they were marginally not significantly different (*p* = 0.078). ATI cytology is a useful diagnostic test in dermatophytosis, in dogs with kerion as well as cats.

## 1. Introduction

Dermatophytosis is a common superficial fungal skin disease that is zoonotic and affects multiple animal species, especially dogs and cats [1,2]. Dermatophytes are fungi that degrade keratin, resulting in hair, skin, and claw disease [2,3]. The most common dermatophytes diagnosed in dogs and cats are *Microsporum canis* (*M. canis*), *Microsporum gypseum* (*M. gypseum*) [new name, *Nannizzia gypsea* (*N. gypsea*)], and *Trichophyton mentagrophytes* (*T. mentagrophytes*) [3,4,5]. *M. canis* is the most frequently isolated species, with a prevalence of >90% in cats and 70–80% in dogs [1].

Diagnosis of dermatophytosis is a combination of compatible history and clinical signs, along with the confirmation of an active infection with the fungi [3]. The tests used for in-clinic diagnosis are direct microscopic examination and fungal culture [2,3]. The most commonly used sample collection technique for direct microscopic examination is plucking hairs. Occasionally, diagnosis requires Wood’s lamp examination as a screening tool to collect infected material. Wood’s lamp can be positive in most cases of *M. canis* dermatophytosis [3,6]. Depending on the study, a positive fluorescence in untreated animals varied from 72 to 100% [3]. Fluorescence is due to the presence of pteridine in the cortex or medulla of the infected hair shaft, thus true fluorescence occurs along the hair shaft and never in scales. It is not associated with the presence of spores or hyphae; therefore, it can still be positive in the tips of hairs in animals that are cured [3]. Skin scrapings can also provide confirmation of dermatophyte infection, and other studies have compared the two sampling techniques [7,8]. In one study, positive results were obtained in 54.1% (20/37) of dogs and 67.5% (27/40) of cats with hair plucks, while skin scraping yielded positive results in 78.4% (29/37) of dogs and 80% (32/40) of cats [8]. In kerion cases, sensitivity of hair plucks and skin scraping is 34.8% (8/23) and 52.2% (12/23), respectively, while impression smear cytology is positive in 91% (21/23) of the cases [7]. Samples from *M. gypseum* (new name *N. gypsea*) and *T. mentagrophytes* infections are usually negative with direct microscopy [8,9]. Fungal culture in Sabouraud’s agar or dermatophytes test medium (DTM) is a very sensitive method for detecting dermatophytes and determining their species, but false positives and false negatives also occur [3]. Point-of-care dermatophyte test medium cultures (PoC-DTM) can be 97% accurate in diagnosing dermatophytosis by clinicians in a referral clinic when compared with diagnostic laboratories [10]. Histopathology is mostly used in kerions, mycetomas, pseudomycetomas, pustular dermatophytosis, and in atypical presentations [3]. The use of a polymerase chain reaction (PCR) to detect dermatophyte DNA can help in achieving a rapid diagnosis [6,11]. However, since it detects both viable and nonviable fungal DNA, a positive result does not always indicate active infection and results must be interpreted in association with clinical signs [3]. In the absence of clinical signs, positive PCR or culture results may indicate the presence of spores on the animal’s coat rather than an infection. These animals can be a source of infection; thus, environmental measures should be taken. No single diagnostic test is the “gold standard” for the diagnosis of dermatophytosis, and diagnosis is made by complementary methods to indicate active infection [3].

Adhesive tape preparations have been used for diagnosis and monitoring the response to therapy in veterinary dermatology [12,13]. Their usefulness for the identification of yeasts, bacteria, and inflammatory cells has been reported [14,15,16,17,18]. Direct microscopy of adhesive tape impressions without staining has also been used in the diagnosis of superficial parasitic infestations [19,20,21]. Moreover, in a book on skin cytology, adhesive tape impressions have been mentioned as the best sampling method when alopecia, scales, erythema, and broken hairs are present, as it is not so uncommon to detect arthroconidia and/or hyphae on corneocytes, especially in cats [18]. To the best of the authors’ knowledge, there are no published studies on the use of ATI cytology for the diagnosis of dermatophytosis, with the exception of one [13]. In that study, fungal elements were found on ATI cytology in all 10 of 10 cats and 8 of 10 dogs that were included. The authors routinely use ATI cytology to examine lesions suspected for dermatophytosis in both dogs and cats.

The aim of the present study was to evaluate the sensitivity of ATI cytology to detect dermatophytes from lesional skin in dogs and cats and to compare the three tests (hair plucks, ATI cytology, and fungal culture) for the diagnosis of canine and feline dermatophytosis.

## 2. Materials and Methods

### 2.1. Study Population and Diagnostic Tests

Thirty client-owned dogs and fifteen client-owned cats diagnosed with dermatophytosis were enrolled in the study. The samples were collected during the routine diagnostic procedure of skin disease, so additional consent from the animals’ owners was not required. Direct examination of plucked hairs and ATI cytology as well as fungal cultures (DTM) were performed in all animals. Briefly, hairs from the periphery and the center of the lesions were plucked with forceps and placed with a drop of mineral oil on a slide. For tape preparations, a clear acetate tape was firmly impressed three times (Scotch^®^ Crystal tape) onto the surface of the lesions and then placed onto a microscope slide over a few drops of blue Diff-Quick stain (Hemacolor^®^, Merck; Darmstadt, Germany). Fungal culture was performed with a DTM (Dermakit Agrolabo, Scarmagno, Italy), and hairs were obtained from the periphery and the center of the lesions with a sterilized forceps following drying of the area after disinfection with an alcohol-moistened gauze. Identification of the dermatophyte species was done with Roth’s flag. 

### 2.2. Statistical Analysis

Six different datasets were used in the statistical analysis to evaluate the effectiveness of the three diagnostic methods (i.e., hair plucks, ATI cytology, and fungal culture) in different animal groups. The different datasets included animals positive to dermatophytosis sorted into cats (*n* = 15), dogs (*n* = 30), cats and dogs (*n* = 45), cats and dogs with alopecia (*n* = 34), dogs with alopecia (*n* = 19), and dogs with kerion (*n* = 11). The ability of each test to correctly identify animals with dermatophytosis was estimated using the sensitivity of the diagnostic test with the corresponding 95% Confidence Interval (C.I.). Subsequently, the sensitivities of the tests were compared using Mc Nemar’s statistical test on paired data, as the same animal individuals were used in the diagnostic tests. Furthermore, the percentage of agreement and the Cohen’s kappa statistic (κ) were used to compare the results of the diagnostic tests in pairs. Finally, the different effectiveness of each test within crucial groups of animals with same characteristics (dogs with alopecia vs. dogs with kerion, dogs with alopecia vs. cats with alopecia) was detected using a Chi-square test of independence or Fisher’s exact test depending on the frequencies of the contingency table. The statistical analysis was implemented using the R package version 3.2.2 [22], while the level of significance of testing was set to 0.05.

## 3. Results

### 3.1. Dogs

Data regarding the signalment of the dogs are reported in Table 1. 

**Figure 1:**
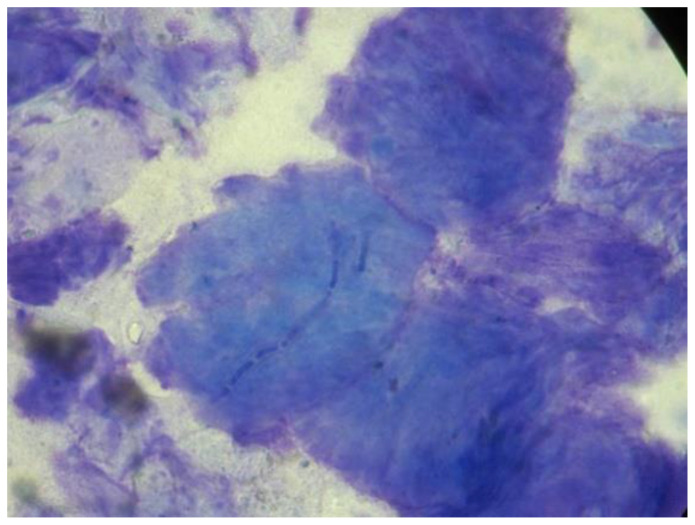
Adhesive tape impression cytology from a dog with dermatophytosis showing a hypha on the surface of a corneocyte (100×).

The results of the diagnostic tests of the 30 dogs are presented in Table 2. In nine dogs all three tests (9/30, 30%) were positive, in sixteen dogs two of the tests (16/30, 53.3%) were positive, and in five dogs only one (5/30, 16.7%) test was positive. The sensitivity of each diagnostic test with the corresponding 95% confidence interval (C.I.) within different animals’ groups is presented in Table 3 Hyphae and spores were seen in 78.3% (18/23) and 65.2% (15/23) of the positive adhesive tape preparations, respectively (Figure 1). *M. canis* was isolated in 95.7% (22/23) of the positive fungal cultures, while *M. canis* and *T. mentagrophytes* were isolated in one case (1/23, 4.3%).

### 3.2. Cats

Data regarding the signalment of the cats are reported in Table 4. 

**Figure 2:**
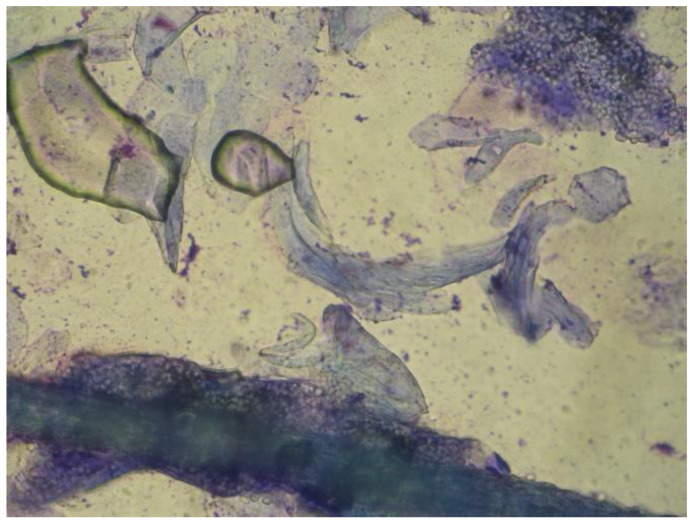
Adhesive tape impression cytology from a cat with dermatophytosis, showing an infected hair with hyphae surrounded by several spores and a cluster of spores on the background (40×).

The results of the diagnostic tests of the fifteen cats are presented in Table 5. In ten out of fifteen cats (66.7%) all three tests were positive, in the remaining five cats four of them had two tests (4/15, 26.7%) positive, and in only one case (6.7%) only DTM was positive. Hyphae and spores were seen in 92.9% (13/14) and 85.7% (12/14) of the positive adhesive tape preparations, respectively (Figure 2). *M. canis* was isolated in 92.3% of the positive fungal cultures (12/13), while *T. mentagrophytes* was isolated in one case (1/13, 7.7%). The sensitivity of each diagnostic test with the corresponding 95% confidence interval (C.I.) within different animals’ groups is presented in Table 3.

### 3.3. Statistical Analysis Results

The three diagnostic tests were compared in pairs for their agreements of the offered results (Table 6). The identification results of the hair plucks showed moderate agreement with both adhesive tape preparations and fungal culture, since the percentage of agreement was around 50% for most of the cases. This was in accordance with the computed low values of the κ statistic within all test comparisons, showing independence among test results (i.e., low intertest reliability). Nevertheless, the results in cats’ datasets seemed more robust, with higher percentages of agreement and values of the κ statistic between tests.

The effectiveness of identifying the affected animals among tests was tested in terms of sensitivity (Table 6, lines of the P-McNemar Test). The statistical analysis showed no significant difference between the three diagnostic tests (*p*-values > 0.05) for all datasets except for the dogs with kerion. In that group, the sensitivity of hair plucks was significantly lower than that of fungal culture (DTM) (*p*-value = 0.041), while the sensitivity of ATI cytology was marginally not significantly different from hair plucks’ sensitivity (*p*-value = 0.078).

Each diagnostic test was tested for its sensitivity between dogs with kerion and dogs with alopecia. The effectiveness of hair plucks was found to be significantly lower in dogs with kerion than in dogs with alopecia (*p* = 0.044). On the other hand, the other two tests (fungal culture and ATI cytology) showed similar effectiveness within the same groups of dogs (*p*-value > 0.05). 

Dogs and cats with alopecia were further combined and tested to reveal possible differences in the tests’ efficiency within these groups of animals. Although the sensitivity was recorded higher in cats than dogs for all tests (80% vs. 73. 7%, 86.7% vs. 68.4%, and 93.3% vs. 68.4% for cats and dogs for hair plucks, fungal culture (DTM), and adhesive tape impression cytology, respectively), the statistical analysis showed no difference in the efficiency of the tests within these groups (*p*-value > 0.05). The result was marginal (*p* = 0.074) for the adhesive tape preparations.

## 4. Discussion

*M. canis* was isolated in most of the cases in this study; the dogs and cats that participated were from urban areas and some of them were recently adopted strays. This correlates with previous studies in which *M. canis* has been reported as the most prevalent species in house pets, namely dogs and cats [23,24,25,26,27,28,29]. Moreover, in a recent paper, the majority of kittens under six months of age that were infected with *M. canis* were either strays, adopted from shelters, or bought from pet shops [26]. Only one cat was positive for *T. mentagrophytes*, while one dog had a mixed infection from *M. canis* and *T. mentagrophytes*. Simultaneous infection with two or more dermatophytes has been reported in other studies [1,14,24,27,30]. The kitten was a recently rescued stray, so it may have come in contact with rodents. The dog was recently adopted from a shelter, so previous contact with rodents, cats, or infected dogs cannot be excluded. All kerions were due to *M. canis*, a finding that correlates with previous studies conducted in the Mediterranean [7,31,32], but in contrast to other studies that report *M. gypseum* (new name *N. gypsea*), *T. mentagrophytes*, and *T. verrucosum* as most common causes of kerion [1,9].

In the present study, one test could be negative and others could be positive, and no test was 100% positive in the diagnosis of dermatophytosis. A large difference in test percentages was seen in dogs with kerion, and this group had the lowest percentage in one diagnostic test: the microscopic examination of plucked hairs. This agrees with a previous study of kerion cases with 34.8% sensitivity of hair plucks and 91% sensitivity of impression smear cytology [7]. The highest percentage of agreement between tests was found in cats. It can possibly be attributed to an increased fungal load on kittens’ and cats’ haircoats and to an outbreak of infection due to skin microtrauma. In this study, most of the feline patients were kittens, and skin traumas in this group can occur, as queens overzealously groom their kittens with their keratinous tongue papillae, or during fighting or playing with their littermates [33]. Additionally, kittens have reduced grooming tendencies and therefore the fungal elements are not mechanically removed, leading to an increase in fungal elements. Another possible explanation is that more cats had multifocal lesions compared to dogs and sampling material may have been more abundant. Most of the dogs and cats were positive for dermatophytosis in at least two diagnostic methods and six of them (five dogs and only one cat) had just one test positive. No conclusions can be made concerning the sensitivity of tests, since in these six cases positive results were equally distributed between the three diagnostic tests. Possible reasons for false negative tests that have been proposed are previous antifungal therapy, poor sampling technique, contaminants, decreased amount of material, and poor incubation conditions [3,10]. In the present cases, mechanical removal of infectious elements through a recent haircut and topical therapy were recognized as potential causes for false negative results. It is therefore evident that according to the type of lesions, appropriate techniques should be used to collect an appropriate sample, and more than one test should be performed. Dermoscopy may be a useful clinical tool, with or without concurrent use of a Wood’s lamp, to identify hairs for culture and/or direct examination [3,34,35].

In a WAVD clinical consensus paper on dermatophytosis in small animals, after a review of the literature, it is concluded that no single diagnostic test can be identified as the “gold standard” for the diagnosis of dermatophytosis [3]. The present study supports this conclusion, since in comparisons made for the sensitivity of diagnostic tests in dogs and cats no significant difference was found, apart from dogs with kerion. A possible explanation for this is that since kerions can be severely inflamed, the fungi cannot persist, and the fungal load is decreased and not easily identified with direct microscopy of hair shafts [36]. Moreover, in the kerion cases alopecia is a common early finding due to severe inflammation, and thus hairs for direct microscopy are obtained from the periphery of the lesions where infection is newly spreading, and where fungal elements are lower in number and hard to be found microscopically. On the contrary, an adequate sample of infected material might be enough in ideal environmental conditions for a successful culture, thus explaining the difference in sensitivity between culture and hair plucks. Moreover, the properties of the tape enable the collection of sufficient material (hair shaft fragments, infected hairs that easily fall out, corneocytes, exudative or seborrheic material) potentially highly informative for diagnosis. Although not statistically significant, adhesive tape preparations were marginally more sensitive in cats than in dogs with alopecia. A possible hypothesis for this is that cats are asymptomatic carriers of *M. canis*, so when they exhibit clinical signs of infection, the fungal load on lesional sites may be higher.

Adhesive tape impression cytology is a diagnostic test that is convenient and rapid to perform, inexpensive, noninvasive, safe, and quick to evaluate [14,18]. Moreover, it is an enhanced collection technique due to the adhesion properties of the tape that are suitable to collect material from both exudative and seborrheic lesions, and the stain is useful to more clearly identify pathogens and cells. In a recent study, fungal spores were detected in all adhesive tape preparations from cats and in none of the canine cases, while hyphae were present in some of the feline samples and in 80% of the canine ones [13]. These results partially correlate with the findings of this study. A difference noted is that in the present study spores were detected in 65.2% of the positive canine adhesive tape preparations, compared to none of the former study. However, the small number of the samples in the other study might have contributed to this difference. Impression smears have been reported sensitive for 91% cases of kerion in dogs [7]. In that study, cytology was superior to fungal culture in sensitivity, 91% versus 74%, respectively, but in our study fungal culture and adhesive tape preparations shared the same sensitivity percentage, 90.9%. Nonetheless, cytology is a very sensitive method regarding kerion diagnosis and should be performed in these cases, as culture may sometimes be negative, direct microscopic examination of plucked hairs is positive in half or fewer of cases, and histopathology might be needed for final diagnosis [3,7].

## 5. Conclusions

Rapid diagnosis of dermatophytosis is essential to prevent further transmission to owners and cohabitating animals. None of the diagnostics tests are identified as the “gold standard,” so a combination of at least two tests, direct microscopic examination of hair and scales and fungal culture, should be used for diagnosis. However, even though microscopic examination of plucked hairs can be useful in diagnosis, in this study it is found that it is less sensitive in dogs with kerions. On the contrary, in kerions, adhesive tape impression cytology is a valuable diagnostic test. Additionally, adhesive tape preparations can be superior to other diagnostic tests in cats. The adhesive tape impression cytology is easy to perform, inexpensive, noninvasive, and minimally stressful for the animals. It can be routinely used as an in-house diagnostic test for dermatophytosis, and from this study’s results, it is suggested in dogs with kerion and in cats. However, more studies with larger populations should be conducted to further evaluate the sensitivity of this diagnostic test in dermatophytosis.

## Figures and Tables

**Table 1:** Signalment data of the 30 dogs with dermatophytosis.

Data	Percentage
*Breed*	
Mixed breed	12/30 (40%)
Boxer	3/30 (10%)
Pitbull	3/30 (10%)
Yorkshire terrier	2/30 (6.7%)
Others	10/30 (33.3%)
*Gender*	
Male	15/30 (50%)
Female	15/30 (50%)
*Age* (median:2, range:1.5–14 y)	
≤1 year	13/30 (43.3%)
1–2 Years	3/30 (10%)
>2 years	14/30 (46.7%)

**Table 2:** Results of diagnostic tests in 30 dogs with dermatophytosis. (A = Alopecia, K = Kerion, ND = Not done).

Case	Clinical Form	Hair Plucks	Adhesive Tape Impression Cytology	Fungal Culture (DTM)
1	A	Positive	Positive	*M. canis*
2	A	Positive	Negative	Negative
3	A	Positive	Positive	Negative
4	A	Positive	Positive	*M. canis*
5	A	Negative	Positive	Negative
6	A	Positive	Negative	*M. canis*
7	A	Positive	Positive	*M. canis*
8	A	Negative	Positive	*M. canis*
9	A	Negative	Positive	*M. canis* *T. mentagrophytes*
10	A	Positive	Negative	*M. canis*
11	A	Positive	Negative	Negative
12	A	Positive	Negative	*M. canis*
13	A	Positive	Positive	*M. canis*
14	A	Positive	Positive	Negative
15	A	Positive	Positive	Negative
16	A	Positive	Positive	*M. canis*
17	A	Negative	Positive	*M. canis*
18	A	Negative	Negative	*M. canis*
19	A	Positive	Positive	*M. canis*
20	K	Positive	Negative	*M. canis*
21	K	Positive	Positive	*M. canis*
22	K	Negative	Positive	*M. canis*
23	K	Negative	Positive	*M. canis*
24	K	Negative	Positive	*M. canis*
25	K	Negative	Positive	*M. canis*
26	K	Negative	Positive	*M. canis*
27	K	Positive	Positive	*M. canis*
28	K	Negative	Positive	Negative
29	K	Negative	Positive	*M. canis*
30	K	Positive	Positive	*M. canis*

**Table 3:** Sensitivity of each diagnostic test with the corresponding 95% confidence interval (C.I.) within different dogs’ groups (NA = Not available).

Diagnostic Test	Dogs (*n* = 30)	Dogs with Alopecia (*n* = 19)	Dogs with Kerion (*n* = 11)	Cats (*n* = 15)	Dogs and Cats (*n* = 45)	Dogs and Cats with Alopecia (*n* = 34)
Hair plucks	18/30, 60.0% (40.6, 77.3)	14/19, 73.7% (48.8, 90.9)	4/11, 36.4% (10.9, 69.2)	12/15, 80.0% (51.9, 95.7)	30/45, 66.7% (51.1, 80.0)	26/34, 76.5% (58.8, 89.3)
Adhesive tape impression cytology	23/30, 76.7% (57.7, 90.1)	13/19, 68.4% (43.5, 87.4)	10/11, 90.9% (58.7, 99.8)	14/15, 93.3% (68.1, 99.8)	37/45, 82.2% (67.9, 92.0)	27/34, 79.4% (62.1, 91.3)
Fungal culture (DTM)	23/30, 76.7% (57.7, 90.1)	13/19, 68.4% (43.5, 87.4)	10/11, 90.9% (58.7, 99.8)	13/15, 86.7% (59.5, 98.3)	36/45, 80.0% (65.4, 90.4)	26/34, 76.5% (58.8, 89.3)

**Table 4:** Signalment data of the 15 cats with dermatophytosis.

Data	Percentage
*Breed*	
DSH	14/15 (93.3%)
Persian	1/15 (6.7%)
*Gender*	
Male	9/15 (60%)
Female	6/15 (40%)
*Age* (median: 2.5 months, range: 1.5 month–11 y)	
≤6 months	13/15 (86.7%)
>6 months	2/15 (13.3%)

**Table 5:** Results of diagnostic tests in 15 cats with dermatophytosis.

Case	Hair Plucks	Adhesive Tape Impression Cytology	Fungal Culture (DTM)
1	Negative	Positive	*M. canis*
2	Positive	Positive	*M. canis*
3	Positive	Positive	Negative
4	Positive	Positive	Negative
5	Positive	Positive	*M. canis*
6	Negative	Negative	*M. canis*
7	Positive	Positive	*M. canis*
8	Positive	Positive	*M. canis*
9	Positive	Positive	*T. mentagrophytes*
10	Positive	Positive	*M. canis*
11	Positive	Positive	*M. canis*
12	Positive	Positive	*M. canis*
13	Positive	Positive	*M. canis*
14	Negative	Positive	*M. canis*
15	Positive	Positive	*M. canis*

**Table 6:** Comparison of adhesive tape impression cytology with the other diagnostic tests using different measures of agreement (NA = Not Applicable,* corresponds to *p* ≤ 0.05).

Comparison of Diagnostic Tests	Measures of Agreement	Cats (*n* = 15)	Dogs (*n* = 30)	Cats and Dogs (*n* = 45)	Cats and Dogs with Alopecia (*n* = 34)	Dogs with Alopecia (*n* = 19)	Dogs with Kerion (*n* = 11)
ATI cytology vs. Hair plucks	% Agreement	86.67	43.33	57.78	67.65	52.63	23.08
	Cohen’s k	0.444	−0.269	−0.075	0.06	−0.148	NA
	*P*-McNemar Test	0.475	0.332	0.169	1	1	0.078
ATI cytology vs. fungal culture (DTM)	% Agreement	80	66.67	71.11	67.65	52.38	81.82
	Cohen’s k	NA	0.068	0.058	0.06	−0.061	NA
	*P*-McNemar Test	1	0.752	1	1	0.724	0.48
Hair plucks vs. Fungal culture (DTM)	% Agreement	66.67	50	55.56	58.82	52.63	45.45
	Cohen’s k	NA	−0.119	−0.111	−0.144	−0.148	0.108
	*P*-McNemar Test	1	0.302	0.359	0.789	1	0.041 *

## Data Availability

The data analyzed for the study are available from the corresponding author upon reasonable request.
